# Improvement of knowledge-based automatic slice-alignment method for cardiac magnetic resonance imaging

**DOI:** 10.1186/1532-429X-14-S1-P272

**Published:** 2012-02-01

**Authors:** Shuhei Nitta, Tomoyuki Takeguchi, Nobuyuki Matsumoto, Shigehide Kuhara, Kenichi Yokoyama, Masamichi Imai, Rieko Ishimura, Toshiaki Nitatori, Timothy Albert

**Affiliations:** 1 Corporate Research & Development Center, Toshiba Corporation, Kanagawa, Japan; 2 MRI Systems Division, Toshiba Medical Systems Corporation, Tochigi, Japan; 3 Department of Radiology, Kyorin University, Faculty of Medicine, Tokyo, Japan; 4 Advanced Diagnostic Imaging Center, Salinas Valley Memorial Hospital, Salinas, CA, USA

## Background

Automatic slice alignment allows images of the six standard cardiac planes as defined in the SCMR Image Acquisition Protocols to be obtained by simple and quick operation. Our previously reported method can detect these planes using ECG-gated breath-hold axial multislice images [1]. Achieving higher accuracy and greater robustness for variation in clinical images will lead to improved usability and reliability, resulting in easier cardiac MR examinations. To achieve these goals, we have substantially refined our previously reported automatic slice-alignment method. A combination of knowledge-based recognition and image processing techniques is applied to multiple feature point search to reduce errors in automatic detection. Volunteer and clinical data were used to evaluate of the degree of improvement.

## Methods

ECG-gated 2D steady-state free precession (SSFP) axial multislice images were acquired using a 1.5-T MRI scanner (Excelart Vantage^TM^ powered by Atlas, Toshiba Medical Systems) during a single breath-hold. The scanning conditions were TR/TE = 4.2/2.1 and matrix = 198x256. The slice thickness was set to 7 mm with no gaps, resulting in a scanning time of less than approximately 20 s. The positions of the mitral valve, the cardiac apex, the left ventricular outflow tract, the tricuspid valve, and the right ventricular corner are detected to determine the long-axis and three short-axis orientations in order to define the 4-chamber, 2-chamber, and 3-chamber views using the proposed method combined with knowledge-based recognition and image processing techniques. The angular error between the results and manual annotation of the normal vector of each view was measured for three subsets (Table 1). Eighteen Japanese clinical data subsets were scored for diagnostic accuracy by two physicians (1: unacceptable, 2: marginal, but diagnostically useful, 3: good, 4: excellent).

**Table 1:** Number of patients and datasets.

	patients	datasets
Japanese healthy volunteer data	17	37
Japanese clinical data	35	35
American clinical data	18	34
all data	70	106

## Results

The proposed method successfully detected the six planes in 106 datasets (Table 1). The processes were completed in approximately 1.5 s (3.0-GHz CPU), which is twice as fast as the conventional method. The angular error and accuracy scores are shown in Table 2. These results are more accurate than those obtained by the conventional method.

**Table 2:** The average angular errors and accuracy scores of the conventional[1] and proposed method.

	methods	type of datasets	short-axis	4-chamber	2-chamber	3-chamber
angular error[degree]	conventional method[1]	Japanese healthy volunteer data	3.39±1.98	8.74±5.30	8.81±5.74	11.02±6.41
		
		Japanese clinical data	5.21±7.48	10.03±7.01	10.92±8.94	10.87±8.01
		
		American clinical data	5.75±8.69	9.90±8.59	8.83±7.33	9.55±4.94
		
		all data	4.75±6.65	9.54±7.01	9.51±7.42	10.50±6.56
	
	proposed method	Japanese healthy volunteer data	3.39±1.98	5.12±3.44	7.15±4.51	5.46±3.65
		
		Japanese clinical data	4.21±2.91	7.79±4.10	11.50±7.90	6.76±4.66
		
		American clinical data	4.83±3.43	5.90±4.24	9.64±8.25	5.14±3.04
		
		all data	4.12±2.85	6.25±4.05	9.38±7.20	5.79±3.87

accuracy score[scale:1-4]	conventional method[1]	Japanese clinical data(18/35)	3.74±0.55	3.47±0.69	3.82±0.46	3.76±0.54
	
	proposed method	Japanese clinical data(18/35)	3.84±0.37	3.79±0.41	3.82±0.39	3.87±0.34

## Conclusions

We have developed a sophisticated slice-alignment method employing knowledge-based recognition combined with image processing techniques to simplify cardiac scan planning. The experimental results showed that the proposed method can detect the cardiac planes more quickly and accurately than the conventional method and is more robust for data from a variety of ethnic groups.

## Funding

No funding was received for this research.
